# Medical and Physical Intelligence

**Published:** 1802-10-01

**Authors:** 


### MEDICAL AND PHYSICAL INTELLIGENCE.

### [ FOREIGN AND DOMESTIC. ]

Sir John Sinclair, in his Effay on Longevity, wifhes the following Queftions to have an extenfive circulation, and invites all perfons, as far as their experience and obfervation will admit, to anfwer them with minutenefs and accuracy.

1. What is the effect of the climate in which you refide, on the health and longevity of the human race?

2. What form is reckoned moft conducive to health and longevity?

3. Is it found, that being defcended from young and from healthy parents, is effential for good health and old age?

4. Is it found, that health and old age depend much on the difpofition or temper of the individual?

5. Is there any perceptible difference in confequence of fituation of life?

6. What profeffions are reckoned favourable to longevity, or otherwife?

7. Is exercife and moderate labour found neceffary for preferving health and long life?

8. Have the long-lived in general been in the marriage ftate?

9. Have the greateft proportion of the long-lived confifted of males or females?

10. Have their been any inftances of perfons renewing their age, getting new teeth, new hair, &c.?

11. What are the other circumftances tending to promote long-life?

12. What is the effect of diet on health and longevity?

13. What are the effects of clothing?

14. What the effect of habitation, and the difference of living in a town or in the country?

15. What are the effects of habits and cuftoms in regard to early rifing, bathing, regular meals, regular fleep, and in particular, what are thofe minute circumftances on which it is fuppofed that health and longevity principally depend?

16. What are the rules regarding medicine, which are accounted the moft ufeful and falutary?

17. What are the moft remarkable inftances of longevity, and how are they authenticated?

18. What are the rules adopted by thofe who have attained great age?

19. Have any tables of longevity been drawn up in your neighbourhood, bourhood, and how do they agree with the one extracted from Hufeland?

20. Do any additional obfervations or particulars occur to you on the fubject of health or longevity?

---

M. Dupuytren, chief of Practical Anatomy, has prefented to the Medical School at Paris, a girl, aged 2 months 10 days, who came into the world only with a trunk: the fituation of the lower extremities was marked by two fmall depreffions; the place of the fuperior extremities was marked by fmall protuberances, that on the right fide was more developed, fo as to form a fhort arm, On diffection, all the mufcles were found to terminate fhort of the ftump; the humerus on the right fide was complete, and terminated, at its lower extremity, by the ufual articular furfaces. On the left there was found but the half, correfponding to the fcapula; interiorly was found a portion of bone, which fhewed that the formation of the femur was commenced.

We cannot but confider every lufus naturæ worth attention; they hold out lights to direct the Phyfiologift and Pathologift in their inquiries.

---

Paris has lately loft a very learned and ingenious phyfician, Xavier Bichat, a pupil of the celebrated Deffault. He was known by various works of great merit, and he is extremely regretted in this country. He has publifhed a book much efteemed, and the only one of the kind yet exifting in Phyfiology, *On Life and Death.* He was preparing for the prefs a new edition with confiderable improvements. He is alfo the author of a *Treatise on Membranes,* wherein they are claffed in a new manner. He publifhed 4 volumes on *general,* and has written two on *descriptive,* Anatomy. It is believed that the two laft on this branch of fcience are now in prefs. When he died, he was preparing a work on *Pathological Anatomy.* Befides thefe original works, he has given a new edition, enriched with many alterations and additions, of fome works of his mafter Deffault, viz. *Diseases of the Urinary Passages; Surgical Journal;* and *Surgical Works.* In confideration of his merit and excellence, Bonaparte, in a letter, dated Thermidor 14, year 10, requefts Chaptal, Minifter of the Interior, to caufe a marble to be erected in the Hotel Dieu, to the memory of Deffault and Bichat, "On account, fays he, of the fervices they have rendered; the firft, to French Surgery, of which he is the reftorer; the fecond, to Phyfic, which he enriched with many ufeful works. Had not Bichat fallen, at twenty-eight years of age, a victim to mercilefs death, he would have extended the dominion of Phyfic, that Science fo important and fo beneficial to humanity."

---

Mr. Read has invented a pneumatic apparatus, the whole of which may be made for lefs than a guinea, and may be introduced to the bed-fide of the patient, who, by turning a cock, may take an infpiration of the gas as often as he pleafes.

M. Da-

M. Damart, of St. Omer, propofes a new method of purifying vegetable oil. He mixes with 100lb. of oil 25 oz. of rock alum, diffolved in 9lb. of boiling water, then ftirring it for about half an hour, he adds 15 oz. of nitric acid, ftill continuing to ftir it, and after it has ftood 48 hours, the pure oil fwims on the furface, and may be drawn off.

---

Profeffor Robison is about to publifh a Chemical Lecture of the late celebrated Dr. Jofeph Black, Regius Profeffor of Chemiftry of Edinburgh, from the author's manufcripts, with Notes, philofophical and hiftorical, by the Editor; partly to illuftrate the Text, and partly to afcertain the claims of Dr. Black, and other eminent philofophers of thefe kingdoms, to the great Difcoveries and Improvements which have been made in Chemiftry fince the Year 1756.

---

Propofals are circulated for publifhing by Subfcription, (for the benefit of the Author's Children) A Courfe of Lectures on Zoonomia, or the Laws of Animal Life, by the late Thomas Garnet, M. D. formerly Profeffor of Natural Philofophy and Chemiftry in the Royal Inftitution of Great Britain; dedicated, by permission, to the Right Honourable and Honourable the Managers of that Corporation; and allowed to be printed at their prefs. In this Courfe a popular view is given of the Animal Economy, and the laws by which its different functions are regulated, with the methods of preventing and curing difeafes. The principal object has been to render this Courfe interefting, not only to ftudents of medicine, but to all who think the ftudy of the human frame a fubject worthy their inquiry. The Courfe confifts of fourteen Lectures, and will form one handfome quarto volume.

Subfcriptions are received by Meffrs. Bofanquet and Co. 73, Lombard-ftreet; Meffrs. Coutts and Co. 59, Strand; and by Mr. Savage, at the houfe of the Royal Inftitution, Albemarle-ftreet.

---

Theatre, London Hospital.

Mr. Headington and Mr. Frampton will commence their Lectures on Anatomy, Phyfiology, and the Principles and Operations of Surgery, on Friday the 1ft of October, at nine in the morning.

Anatomical Demonftrations and Diffection, by Mr. Armiger.

During the Winter, a Series of Lectures on Surgery, illuftrated by cafes under treatment, will be given to the pupils of the Hofpital by Mr. Blizard, Mr. T. Blizard, and Mr. Headington.

Dr. Dennison will alfo commence his Lectures on the Theory and Practice of Midwifery, on the 1ft of October, at eleven in the forenoon.

---

Dr. James Curry, Affiftant Phyfician to Guy's Hofpital, has been unanimoufly elected one of the Senior Phyficians to faid Eftablifhment, in the room of Dr. Harvey, refigned.

Dr.

Dr. Jameson's Clinical Lectures will commence on the 12th of October, at the Finſbury Diſpenſary, St. John's Square; and a Lecture will be delivered on Tueſday, Thurſday, and Saturday in every week, between the hours of 11 and 12 o'clock; one of theſe will be on the Principles, and the other on the Practice of Medicine, alternately: After each Lecture, an hour and an half will be ſet apart to examine the patients of the Diſpenſary.

---

Dr. Bradley (removed from Delahay-ſtreet to Parliament-ſtreet) will commence his Autumnal Courſe of Lectures on the Theory and Practice of Medicine, on Monday, October 4, at the Lecture Room, No. 102, Leadenhall-ſtreet, at ſix in the afternoon.

---

Mr. Thomas will commence his Winter Courſe of Lectures on the Principles and Operations of Surgery, on Friday the 8th of October, at ſeven o'clock in the evening.

Further particulars may be known at his houſe in Leiceſter Square, or at the Anatomical Theatre, Windmill Street.

